# Brominated Selinane Sesquiterpenes from the Marine Brown Alga *Dictyopteris divaricata*

**DOI:** 10.3390/md7030355

**Published:** 2009-07-22

**Authors:** Nai-Yun Ji, Wei Wen, Xiao-Ming Li, Qin-Zhao Xue, Hua-Ling Xiao, Bin-Gui Wang

**Affiliations:** 1 Yantai Institute of Coastal Zone Research for Sustainable Development, Chinese Academy of Sciences, Yantai 264003, China; E-Mails:wenwei_500@sina.com (W.W.);qzxue@yic.ac.cn (Q-Z.X.); 2 Institute of Oceanology, Chinese Academy of Sciences, Qingdao 266071, China; E-Mail:lixmqd@yahoo.com.cn (X-M.L.); 3 Ocean University of China, Qingdao 266003, China; E-Mail:backofangel@hotmail.com

**Keywords:** Dictyopteris divaricata, sesquiterpene, selinane

## Abstract

Two new brominated selinane sesquiterpenes, 1-bromoselin-4(14),11-diene (**1**) and 9-bromoselin-4(14),11-diene (**2**), one known cadinane sesquiterpene, cadalene (**3**), and four known selinane sesquiterpenes, *α*-selinene (**4**), *β*-selinene (**5**), *β*-dictyopterol (**6**), and cyperusol C (**7**), were isolated from a sample of marine brown alga *Dictyopteris divaricata* collected off the coast of Yantai (China). Their structures were established by detailed MS and NMR spectroscopic analysis, as well as comparison with literature data.

## 1. Introduction

The marine brown alga *Dictyopteris divaricata*, found widely spread along the coasts of China, has provided many structurally interesting sesquiterpenes and norsesquiterpenes [1–4]. However, to date there have been no reports of halogenated structures isolated from this species. Our continuing investigation of the secondary metabolites of *D. divaricata* collected off the coast of Yantai has revealed two new brominated sesquiterpenes, 1-bromoselin-4(14),11-diene (**1**) and 9-bromoselin-4(14),11-diene (**2**), one known cadinane sesquiterpene, cadalene (**3**) [5], and four known selinane sesquiterpenes, *α*-selinene (**4**) [6], *β*-selinene (**5**) [6], *β*-dictyopterol (**6**) [7], and cyperusol C (**7**) [8]. The isolation and structural determination of compounds **1–7** are the subject of this paper.

## 2. Results and Discussion

**Figure 1:**
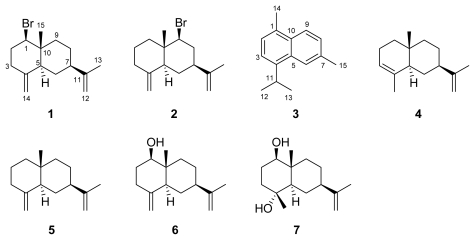
Structures of compounds **1–7**.

The dried and powdered alga *D. divaricata* was extracted with the mixture of CHCl_3_ and MeOH (1:1, v/v). The concentrated extracts were partitioned between H_2_O and EtOAc. The EtOAc-soluble fraction was purified by a combination of silica gel, reversed-phase silica gel, and Sephadex LH-20 column chromatography, as well as preparative TLC procedure, to yield compounds **1–7** (Figure 1).

Compounds **1–3** were obtained as a colorless oily mixture. They displayed one spot by TLC analysis (*Rf* 0.65, petroleum ether), and all attempts to separate them failed due to their low polarity. The IR spectrum showed absorptions of vinyl and phenyl groups at *v*_max_ 1643, 1604, 1512, and 1450 cm^−1^ and no absorption of hydroxyl groups. The GC-EIMS gave three peaks with molecular ion peaks at *m*/*z* 198, 282/284 (1:1) and 282/284 (1:1) [M]^+^, which indicated that the mixture was composed of three compounds. The ^1^H- and ^13^C-NMR data of these three compounds **1–3** could be distinguished with the aid of the NMR data shown in Table 1.

The compound with the molecular ion peak at *m*/*z* 198 was tentatively identified as cadalene (**3**) by comparison with the EIMS database [5]. Fortunately, the ^1^H and ^13^C-NMR peaks of **3** could be found in NMR spectra (Table 1). H-2 was *ortho* to H-3 and H-8 was *ortho* to H-9 according to their large coupling constants (*J* > 7 Hz). The coupling constant 1.7 Hz between H-6 and H-8 indicated H-6 was *meta* to H-8. The above connections were corroborated by the ^1^H-^1^H COSY correlations between H-2/H-3, H-6/H-8, H-8/H-9. The presence of **3** was further confirmed by the observed HMBC correlations from H-11 to C-3, C-4, C-12, and C-13, from H-12 to C-4, C-11, and C-13, from H-13 to C-4, C-11, and C-12, from H-14 to C-1, C-2, and C-10, and from H-15 to C-6, C-7, and C-8. It is obvious then that the molecular ion clusters at *m*/*z* 282/284 (1:1) [M]^+^ correspond to the other two compounds **1–2**, whose molecular formulae were determined as C_15_H_23_Br on the basis of HRESIMS (*m*/*z* 203.1784 [M–Br]^+^, calcd. for C_15_H_23_Br, 203.1799).

The ^1^H-NMR spectrum of **1** displayed two methyl singlets, one double-doublet assignable to a halogenated/oxygenated methine, and four broad characteristic olefinic protons singlets. The ^13^C-NMR spectrum along with the DEPT and HSQC experiments revealed the presence of two methyls, seven methylenes, three methines, and three quaternary carbon atoms. A detailed comparison of the NMR data with those reported for *β*-selinene (**5**) and *β*-dictyopterol (**6**) revealed the similarity between them [6,7], although n contrast to *β*-selinene (**5**) and *β*-dictyopterol (**6**), C-1 in **1** was brominated [*δ*_C_ 67.9 (d)], as established by comparison with the reported data for 1-bromo-4-hydroxyselin-7-ene [9]; this was corroborated by the molecular ion cluster at *m*/*z* 282/284 (1:1) [M]^+^ and fragment ion peak at *m*/*z* 203 [M–Br]^+^ in the EIMS and the molecular formula C_15_H_23_Br. The observed HMBC correlations from H-12 to C-7 and C-13, from H-13 to C-7, C-11, and C-12, from H-14a and H-14b to C-3 and C-5, and from H-15 to C-1, C-5, C-9, and C-10 confirmed the planar structure of **1**.

The relative configuration of **1** was determined by analysis of coupling constants and NOESY correlations, as well as by comparison with literature data. H-1 was axial, as indicated by its large coupling constant (11.8 Hz), and located on the same face of H-5 from the NOESY correlation between them. The NOESY correlations between H-15/H-2a, H-6a, H-8a indicated C-15, H-2a, H-6a, and H-8a to be axial and the decalin ring to be *trans*-fused. C-11 was equatorial and the configuration at C-7 was the same as in **4–7** based on their identical NMR data and the observed NOESY correlation between H-7/H-9a [6–8]. The above evidence established the structure of **1** as 1-bromoselin-4(14),11-diene. Compound **1** has also been isolated in pure form from *Laurencia pinnata*, collected off the coast of Nanji Island (China), and its structure was confirmed unambiguously by 1D/2D NMR and HRAPPIMS (high-resolution atmospheric pressure photoionization mass spectroscopy), and this data was used to deconvolute the spectra of the **1–3** mixture [10].

The ^1^H-NMR spectrum of **2** also displayed two methyl singlets, one double-doublet attributed to a halogenated/oxygenated methine, and four broad singlets ascribable to olefinic protons. The ^13^C-NMR spectrum, along with the DEPT and HSQC experiments, revealed the presence of two methyls, seven methylenes, three methines, and three quaternary carbon atoms. Based on their identical molecular ion clusters at *m*/*z* 282/284 (1:1) [M]^+^ and fragment ion peaks at *m*/*z* 203 [M–Br]^+^ in the EIMS and the molecular formulae C_15_H_23_Br, compound **2** should be an isomer of **1**, a fact conformed by analysis of their NMR data. The NMR data were also compared with those reported for *β*-selinene (**5**), showing the similarity between them [6]. In contrast to *β*-selinene (**5**), C-9 at *δ*_C_ 52.2 (d) was brominated in **2**, as indicated by the HMBC correlation from H-15 to C-9. The other HMBC correlations from H-12 to C-7 and C-13, from H-13 to C-7, C-11, and C-12, from H-14a and H-14b to C-3 and C-5, and from H-15 to C-1, C-5, and C-10 confirmed the structure of **2**. The relative configuration of **2** was determined by analysis of coupling constants and NOESY correlations, as well as by comparison with literature data. H-9 was axial, as suggested by its large coupling constant (12.0 Hz), and, based on the NOESY correlation between them, located on the same face as H-5. The decalin ring was *trans*-fused based on the identical chemical shifts of C-5 and C-10 in **2** and **1**. The C-11 was equatorial and the configuration at C-7 was identical with those of **1**, **4–7** according to their similar NMR data [6–8]. The above evidence established the structure of **2** to be 9-bromoselin-4(14),11-diene.

The structures of known compounds **4–7** were confirmed by detailed NMR data comparison with those in the literature [6–8]. To the best of our knowledge, compounds **1** and **2** represent the first example of halogenated terpenes from the genus *Dictyopteris*, and are new additions to the molecular diversity of this genus. Compounds **1** and **2** may act as effective chemical defenses against marine herbivores by comparison with similar brominated structures [11].

## 3. Experimental Section

### 3.1. General

NMR spectra were recorded in CDCl_3_ at 500 and 125 MHz for ^1^H and ^13^C, respectively, on a Bruker Avance 500 MHz NMR spectrometer with TMS as internal standard. GC-EIMS spectra were determined on a Thermo Scientific ITQ 900 spectrometer. High resolution mass spectra were measured on a VG Autospec 3000 mass spectrometer. IR spectra were obtained on a JASCO FT/IR-4100 Fourier Transform Infrared spectrometer. Column chromatography was performed with silica gel (200–300 mesh, Qingdao Haiyang Chemical Co., Qingdao, P.R. China), RP-18 reversed-phase silica gel (ODS-A, YMC), and Sephadex LH-20 (Pharmacia). TLC was carried out with precoated silica gel plates (GF-254, Qingdao Haiyang Chemical Co., Qingdao, P.R. China). All solvents were of analytical grade.

### 3.2. Algal Material

The brown alga *Dictyopteris divaricata* was collected off the coast of Yantai (lat. 37°31′15″N, long. 121°26′59″E), Shandong Province, P. R. China, in July 2008, and a voucher specimen (MBA0807) has been deposited at the Bio-Resource Laboratory of Yantai Institute of Coastal Zone Research for Sustainable Development, Chinese Academy of Sciences.

### 3.3. Extraction and Isolation

Dried and powdered alga *D. divaricata* (2 kg) was extracted with a 1:1 (v/v) mixture of CHCl_3_ and MeOH. The concentrated extract was partitioned between H_2_O and EtOAc. The EtOAc-soluble fraction (90 g) was fractioned by silica gel column chromatography [petroleum ether (PE)/EtOAc gradient] to give ten fractions, I-X. Fraction I, eluted with PE, was further purified by silica gel column chromatography (PE) to afford a mixture of **1–3** (10.0 mg) and a mixture of **4** and **5** (40.9 mg). Fraction VII, eluted with PE/EtOAc (2:1), was further purified by Sephadex LH-20 (CHCl_3_/CH_3_OH) and RP-18 (CH_3_OH/H_2_O 3:1) column chromatography and preparative TLC (PE/EtOAc 3:1) to afford **6** (5.1 mg). Fraction IX, eluted with EtOAc, was further purified by Sephadex LH-20 (CHCl_3_/CH_3_OH) and RP-18 (CH_3_OH/H_2_O 9:1) column chromatography and preparative TLC (EtOAc) to afford **7** (5.5 mg).

*1-Bromoselin-4(14),11-diene (****1****).* Colorless oil; ^1^H-NMR and ^13^C-NMR: see Table 1; EIMS *m*/*z* (relative intensity): 284/282 [M]^+^ (3), 203 [M–Br]^+^ (98), 161 (26), 159 (28), 147 (88), 133 (36), 119 (52), 105 (89), 91 (100), 79 (54), 77 (46), 67 (37)

*9-Bromoselin-4(14),11-diene (****2****).* Colorless oil; ^1^H-NMR and ^13^C-NMR: see Table 1; EIMS *m*/*z* (relative intensity): 284/282 [M]^+^ (2), 203 [M–Br]^+^ (59), 161 (26), 159 (11), 147 (61), 133 (28), 119 (44), 105 (78), 91 (100), 79 (56), 77 (52), 67 (46).

*Cadalene (****3****).* Colorless oil; ^1^H-NMR and ^13^C-NMR: see Table 1; EIMS *m*/*z* (relative intensity): 198 (59), 183 (100), 168 (48), 153 (16).

IR (KBr) of **1–3**: 3,074, 2,931, 2866, 1,643, 1,604, 1,512, 1,450, 1,377, 887 and 829 cm^−1^.

## Figures and Tables

**Table 1:** ^1^H and ^13^C-NMR data of compounds **1–3** (in CDCl_3_, *δ* in ppm, *J* in Hz).

***No.***	**1**		**2**		**3**	
	*δ*_C_	*δ*_H_	*δ*_C_	*δ*_H_	*δ*_C_	*δ*_H_
1a	67.9 d	4.09 (dd, 11.8, 4.4)	42.8 t	1.14 (m)	131.8 s	
1b				1.91 (m)		
2a	35.0 t	2.15 (m)	27.1 t	1.43 (m)	125.6 d	7.22 (d, 7.2)
2b		2.21 (m)		1.61 (m)		
3a	37.2 t	2.15 (m)	39.1 t	2.17 (m)	121.4 d	7.28 (d, 7.2)
3b		2.32 (m)		2.17 (m)		
4	147.5 s		147.8 s		142.1 s	
5	49.7 d	1.91 (m)	49.1 d	1.99 (m)	131.6 s	
6a	30.1 t	1.41 (m)	30.7 t	1.35 (m)	122.9 d	7.92 (d, 1.7)
6b		1.63 (m)		1.62 (m)		
7	45.4 d	1.95 (m)	45.7 d	1.95 (m)	134.7 s	
8a	26.8 t	1.40 (m)	37.7 t	2.39 (m)	127.2 d	7.35 (dd, 8.5, 1.7)
8b		1.66 (m)		2.39 (m)		
9a	39.8 t	1.18 (m)	52.2 d	4.30 (dd, 12.0, 4.0)	124.8 d	7.92 (d, 8.5)
9b		2.04 (m)				
10	41.2 s		41.0 s		131.2 s	
11	150.0 s		149.9 s		28.3 d	3.72 (h, 7.0)
12a	108.6 t	4.73 (br s)	108.6 t	4.73 (br s)	23.6 q	1.27 (d, 7.0)
12b		4.73 (br s)		4.73 (br s)		
13	21.0 q	1.75 (s)	21.0 q	1.75 (s)	23.6 q	1.27 (d, 7.0)
14a	107.7 t	4.55 (br s)	107.6 t	4.56 (br s)	19.4 q	2.65 (s)
14b		4.78 (br s)		4.73 (br s)		
15	12.0 q	0.85 (s)	14.1 q	0.86 (s)	22.0 q	2.56 (s)
